# SGLT-2-inhibition with dapagliflozin reduces tissue sodium content: a randomised controlled trial

**DOI:** 10.1186/s12933-017-0654-z

**Published:** 2018-01-04

**Authors:** M. V. Karg, A. Bosch, D. Kannenkeril, K. Striepe, C. Ott, M. P. Schneider, F. Boemke-Zelch, P. Linz, A. M. Nagel, J. Titze, M. Uder, R. E. Schmieder

**Affiliations:** 1 Department of Nephrology and Hypertension, University Hospital Erlangen, Ulmenweg 18, 91054 Erlangen, Germany; 2 Department for Diagnostic Radiology, University Hospital Erlangen, Erlangen, Germany; 3 Division of Clinical Pharmacology, Department of Medicine, Vanderbilt University School of Medicine, Nashville, TN USA

**Keywords:** Magnetic resonance imaging, Type 2 diabetes, SGLT-2 inhibition, Tissue sodium content

## Abstract

**Background and aims:**

Sodium tissue content by ^23^Na magnetic resonance imaging (Na-MRI) has been validated in experimental and human studies. SGLT-2 inhibition blocks the reabsorption of glucose and of sodium in the proximal tubular cells in a 1:1 fashion. We hypothesized that SGLT-2 inhibition in patients with type 2 diabetes characterized by sodium retention leads to decreased tissue sodium content due to its pharmacological action.

**Materials and methods:**

In a prospective double blind, placebo controlled, cross-over trial 59 patients (61 ± 7.6 years) with type 2 diabetes were randomized to either dapagliflozin 10 mg or placebo once daily for 6 weeks each. In addition to metabolic parameters and ambulatory blood pressure (BP) we analysed the sodium content in the skin and muscles of the lower leg by Na-MRI.

**Results:**

Compared to baseline 6 weeks treatment with the SGLT-2 inhibitor dapagliflozin decreased fasting (132 ± 28 vs. 114 ± 19 mg/dl, p < 0.001), postprandial blood glucose (178 ± 66 mg/dl vs. 153 ± 46 mg/dl, p < 0.001), body weight (87.6 vs. 86.6 kg, p < 0.001) and systolic (129 ± 12 vs. 126 ± 11 mmHg, p = 0.010), and diastolic (77.4 ± 9 vs. 75.6 ± 8 mmHg, p = 0.024), 24-h ambulatory BP. Tissue sodium content in the skin was reduced after 6 weeks treatment with dapagliflozin compared to baseline [24.1 ± 6.6 vs. 22.7 ± 6.4 A.U.(arbitrary unit) p = 0.013]. No significant reduction of tissue sodium content was observed in the muscle (M. triceps surae: 20.5 ± 3.5 vs. 20.4 ± 3.7 A.U. p = 0.801). No clear significant difference in tissue water content of muscle and skin was observed after 6 weeks of treatment with dapagliflozin, compared to baseline.

**Conclusion:**

SGLT-2 inhibition with dapagliflozin resulted in a significant decrease in tissue sodium content of the skin after 6 weeks. This observation point to a decrease of total sodium content in patients with type 2 diabetes prone to cardiovascular complications, that might be mitigated by SGLT-2 inhibition.

*Trial registration* The study was registered at http://www.clinicaltrials.gov (NCT02383238) retrospectively registered

## Background

Abnormal renal sodium handling is one of the key mechanisms that lead to hypertension and volume overload in diabetic patients, increasing cardiovascular and all-cause mortality. Increased dietary salt intake has been repeatedly found to be associated with cardiovascular damage [1] and, most recently, increased tissue sodium content in the skin was linked to left ventricular hypertrophy [2], independent of blood pressure and other confounders. First evidence has been found that patients with diabetes have increased tissue sodium content in the skin and muscles [3]. Hence, reduction in total sodium content emerged as a therapeutic goal in treating diabetic patients.

Sodium glucose cotransporter 2 (SGLT-2) inhibitors are now widely used for antihyperglycemic therapy. SGLT-2 inhibitors decrease reabsorption of glucose in the renal tubular system. Their unique insulin-independent glycosuric mechanism is characterized by inhibition of glucose reabsorption in the proximal tubular cells, in parallel to blockade of sodium reabsorption. These effects cause osmotic diuresis and natriuresis and decrease systolic and diastolic blood pressures (BP), [4–6] as well as weight loss [7]. Reduction in BP might be related to body weight loss or/and decrease of total body sodium content. Correction of the increased total body sodium content may directly affect the myocardium [2, 8], and potentially lead to reduction of left ventricular hypertrophy. Compensatory sodium reabsorption by other transporter and in the distal tubular system may counteract sodium loss. Indeed, with means of 24-h sodium excretion, exaggerated natriuresis after initiating SGLT-2 inhibition attenuates after 1–2 weeks [6, 9, 10]. However, 24-h sodium excretion must be considered to be highly variable from day to day and is therefore insufficient to reflect true sodium excretion over time [11].

In the last 10 years we and others have developed ^23^Na magnetic resonance imaging (^23^Na-MRI) as a new technique to allow investigations of the role of tissue sodium content in human subjects [12–15]. First studies with ^23^Na-MRI have shown that skin sodium is related to the level of blood pressure in patients with resistant hypertension, confirming experimental findings, that sodium retention and high tissue sodium content have been linked with arterial hypertension in animal models [16, 17]. Furthermore in patients with chronic renal failure and type 2 diabetes both known to retain sodium we observed that tissue sodium content is increased [2, 3].

Dapagliflozin is a SGLT-2 inhibitor and its anti-hyperglycaemic and anti-hypertensive effects have been demonstrated in a number of trials [18]. So far, there are no available data regarding change of tissue sodium content after initiating SGLT-2 inhibition.

The current exploratory study was performed to evaluate the changes of tissue sodium content in patients with type 2 diabetes mellitus after 6 weeks of treatment with dapagliflozin.

## Materials and methods

### Study design

The study was a prospective, randomized, double-blind, placebo-controlled, cross-over phase III b trial performed at the Clinical Research Center of Erlangen-Nuremberg, Germany (http://www.crc-erlangen.de) to characterize microvascular changes in type 2 diabetes. The principal results are reported elsewhere [19].

Patients with type 2 diabetes were recruited from the University outpatient clinic, through physician referrals, and through the use of newspaper advertisements and consecutively enrolled if they fulfilled all inclusion and none of the exclusion criteria. After a run-in/washout period [2 weeks for patients not receiving anti-diabetic treatment (N = 13) and 4 weeks for patients treated with anti-diabetic medication (N = 46), respectively], patients were randomised to receive either once daily oral dapagliflozin 10 mg or placebo through the use of computer generated algorithm for this single centre study. After 6 weeks, there was a 1-week washout period, and then the patients crossed over to the other treatment.

The study was approved by the Ethics Committee of the University of Erlangen (IRB/IEC) on the 7th February 2014. Furthermore, it was performed in accordance with the Declaration of Helsinki. All patients provided written informed consent prior to inclusion in the study. The study was registered at http://www.clinicaltrials.gov (NCT02383238).

### Study population

Patients were included in the study if they had type 2 diabetes and were between 18 and 70 years of age. Individuals were excluded if they had any other form of diabetes, were treated with insulin or more than one oral anti-diabetic drug, were treated with any medication with loop diuretics, had an HbA1c level ≥ 10% (86 mmol/mol), had a fasting plasma glucose (FPG) level > 240 mg/dl, had BP ≥ 180/110 mmHg, had an eGFR < 60 ml/min/1.73 m^2^, or had a body mass index (BMI) > 40 kg/m^2^.

### Endpoints

The primary endpoint of this exploratory substudy was to analyse the effect of dapagliflozin from baseline on the sodium content in the skin and muscles of the lower leg, measured by ^23^Na-MRI. Changes in tissue sodium content in the placebo group from baseline served as a control group. The study was not powered to compare any effect between the groups.

Other clinical characteristics that were measured in parallel were blood glucose levels, HbA1c, office BP and 24-h ambulatory BP.

### Clinical parameters

Demographic data were recorded at the first visit. At the randomisation visit, a fasting blood sample was taken in order to measure HbA1c, FPG, lipid levels, and other biochemical safety parameters (e.g. creatinine, liver enzymes).

To estimate dietary salt intake, a 24-h urine collection for sodium excretion, the most reliable analytic standard [20, 21], was performed.

Office BP and heart rate measurements were taken in a seated position after 5 min of rest. Twenty-four hour ambulatory BP was measured in parallel with Spacelab 90207 (Spacelabs Health Care.WA, USA). Measurements were taken every 15 min throughout the day and every 30 min during the night.

All biochemical, microvascular and macrovascular parameters were re-analysed after each of the two 6-week treatment periods [19]. Any adverse events that occurred during the study were recorded.

## ^23^Na-MRI measurements

Tissue sodium content was assessed noninvasively with a 3.0T clinical MR system (Magnetom Verio, Siemens Healthineers, Erlangen, Germany). ^23^Na-MRI was performed with a gradient echo sequence, total acquisition time: 13.7 min, echo time: 2.07 ms, repetition time: 100 ms, flip angle: 90°, 128 averages, resolution: 3 × 3 × 30 mm^3^) and a frequency-adapted monoresonant transmit/receive birdcage knee coil (32.6 MHz, Stark-Contrast, Erlangen, Germany). ^1^H-water imaging was performed with the body coil of the scanner using a fat suppressed spin-echo sequence (total acquisition time: 6.5 min, echo time: 12 ms, repetition time: 3000 ms, inversion time: 210 ms, resolution: 1.5 × 1.5 × 5 mm^3^) Subjects placed one lower leg in the centre of the ^23^Na knee coil. ^23^Na-MRI measurements of saline solutions with increasing Na^+^ concentration (10, 20, 30, and 40 mmol/l) served to calibrate relative tissue Na^+^. Since our method underestimates the true tissue sodium content, we prefer to use A.U. (arbitrary unit) instead of mmol/l. Additionally, the 10 mmol/l standard defined a relative tissue specific water content of 1 A.U. in the water images. Accuracy of this method has been previously shown [12]. The average coefficient of variation for the analysis of same images between seven different readers was found to be 2.1% for skin sodium, 0.5% for muscle sodium, 7.6% for skin water, and 0.5% for muscle water (inter-reader variability) [2].

### Statistics

Data are presented as absolute values and percentages or means with standard deviation (SD). Statistical significance of differences between baseline and changes due to therapy were determined using a paired t-test. Statistical analysis was performed using SPSS release 19.0.

## Results

### Patients

A total of 67 patients were screened, with 62 undergoing randomisation, 31 to initial dapagliflozin and 31 to initial placebo treatment. Of these, 59 patients completed the study and had all the required MRI data available (full analysis set). The mean age was 60.3 years and 39.0% were female (Table 1). The mean duration of diabetes was 5.54 years and the mean HbA1c level was 6.67% (49 mmol/mol).

**Table 1:** Patients characteristics

	Mean ± SD or n/N (%)
Age (years)	60.3 ± 7.6
Female gender	23/58 (39.0)
Body weight (kg)	87.6 ± 13
Height (cm)	171 ± 11
BMI (kg/m^2^)	29.8 ± 4.3
Mean duration of diabetes (years)	5.54 ± 4.9
HbA1c (%)	6.67 ± 0.7%
Blood glucose concentration	
Fasting (mg/dl)	132 ± 28
Postprandial^a^ (mg/dl)	178 ± 66
Office blood pressure	
Systolic (mmHg)	130 ± 13
Diastolic (mmHg)	80 ± 9.4
Heart rate (bpm)	69 ± 11
24 h ambulatory blood pressure	
Systolic (mmHg)	129 ± 12
Diastolic (mmHg)	77 ± 9
Heart rate (bpm)	74 ± 11
Haematocrit (%)	40.1 ± 2.7
Serum sodium concentration (mmol/l)	138.6 ± 2.0
Urinary sodium excretion over 24 h (mmol/day)	216 ± 81

N = 59. *BMI* body mass index, *HbA1c* glycated haemoglobin

^a^A standardised breakfast was given

After 6 weeks of treatment, HbA1c concentration had not changed significantly from baseline for neither the dapagliflozin nor placebo, related to the short duration of treatment (Table 2). FPG decreased after dapagliflozin treatment by 18 mg/dl (p < 0.001), while FPG after placebo did not change from baseline. Similar finding was observed for postprandial glucose concentrations. Body weight was significantly lower in the dapagliflozin group after 6 weeks of treatment (87.6 vs. 86.6 kg, p < 0.001).

**Table 2:** Clinical characteristics after 6 weeks of dapagliflozin and placebo treatment, respectively

	Placebo Mean ± SD (change from baseline)	p-value vs. baseline	Dapagliflozin Mean ± SD (change from baseline)	p-value vs. baseline
BMI (kg/m^2^)	29.9 ± 4.2 (+ 0.1)	0.846	29.5 ± 4.1 (−0.3)	< 0.001
HbA1c (%)	6.79 ± 0.8 (+ 0.12)	0.064	6.62 ± 0.7 (− 0.05)	0.224
Glucose				
Fasting (mg/dl)	135 ± 32 (+ 2.0)	0.325	114 ± 19 (−18)	< 0.001
Postprandial^†^ (mg/dl)	180 ± 67 (+ 1.0)	0.766	154 ± 46 (− 24)	< 0.001
Office blood pressure				
Systolic (mmHg)	129 ± 13 (− 0.1)	0.340	126 ± 12 (− 4.0)	0.015
Diastolic (mmHg)	79 ± 8.7 (− 1.0)	0.827	78 ± 8.8 (− 2.0)	0.058
Heart rate (bpm)	67.8 ± 9.6 (− 1.3)	0.123	68.2 ± 10.6 (− 0.9)	0.332
24-h ambulatory blood pressure				
Systolic (mmHg)	129 ± 10.8 (− 0.5)	0.172	126 ± 10.8 (−3.0)	0.010
Diastolic (mmHg)	77.1 ± 7.3 (0.0)	0.765	75.4 ± 7.7 (−2.0)	0.024
Heart rate (bpm)	75.7 ± 9.5 (+ 1.4)	0.997	74.1 ± 7.6 (− 0.8)	0.849
Haematocrit (%)	40.3 ± 3.1 (+ 0.2)	0.389	41.1 ± 2.9 (+ 1.0)	< 0.001
Serum sodium conc. (mmol/l)	138.1 ± 1.6 (− 0.5)	0.034	138.3 ± 1.6 (− 0.3)	0.308
Urinary sodium excretion over 24 h (mmol/day)	222.5 ± 103.6 (− 6.0)	0.660	210.1 ± 71.2 (+ 6.5)	0.586

N = 59. *BMI* body mass index, *HbA1c* glycated haemoglobin

^†^A standardised breakfast was given

Office systolic BP decreased by 4 mmHg from baseline after treatment with dapagliflozin (p = 0.015), but only 1 mmHg after placebo (p = 0.340). Diastolic BP was also lower after treatment with dapagliflozin by 2 mmHg (p = 0.058). Twenty-four hour ambulatory BP was reduced, both systolic and diastolic, (p = 0.021 and p = 0.027, respectively), whereas no change was observed in the placebo group.

Serum sodium concentration did not change after dapagliflozin and placebo therapy. Likewise, sodium excretion in 24 h urine as an estimate of daily salt intake did not decrease significantly during dapagliflozin treatment compared to baseline (p = 0.586). The same was found for placebo treatment (p = 0.660). Lipid levels remained stable during the study (data not shown).

### Skin and muscle sodium content after 6 weeks of treatment (Table 3)

**Fig. 1:**
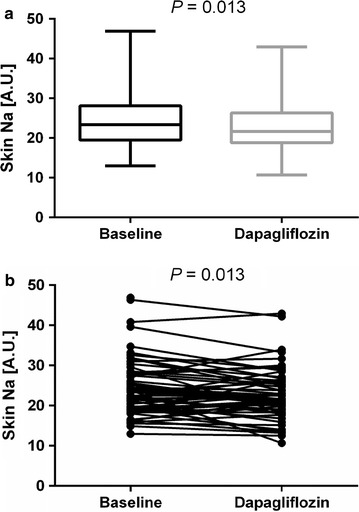
Box and whisker plot (**a**) and linear graphic (**b**) of changes in skin sodium content after 6 weeks treatment with dapagliflozin

Tissue sodium content in the skin was reduced after 6 weeks treatment with dapagliflozin compared to baseline (24.1 ± 6.6 vs. 22.7 ± 6.4 A.U.; p = 0.013) (Fig. 1). No significant reduction of tissue sodium content was observed after 6 weeks of treatment with dapagliflozin in the muscle (M. triceps surae: 20.6 ± 3.5 vs. 20.4 ± 3.7 A.U.; p = 0.801). No significant difference in tissue water content of muscle and skin was observed after 6 weeks of treatment with dapagliflozin, compared to baseline (H_2_O skin: 0.153 ± 0.06 vs. 0.144 ± 0.6 A.U. p = 0.073).

**Table 3:** Skin and muscle sodium and water content at baseline and after 6 weeks of treatment

	Baseline	Dapagliflozin vs. baseline		Placebo vs. baseline	
	X ± SD	X ± SD	p-value	X ± SD	p-value
Skin Na + (A.U.)	24.5 ± 7.2	22.7 ± 6.4	0.013	23.8 ± 8.3	0.314
Skin H_2_O (A.U.)	0.153 ± 0.06	0.144 ± 0.06	0.073	0.15 ± 0.07	0.763
Na + M. triceps surae (A.U.)	20.6 ± 3.5	20.4 ± 3.7	0.801	20.3 ± 3.6	0.514
H_2_O M. triceps surae (A.U.)	0.513 ± 0.03	0.513 ± 0.03	0.952	0.505 ± 0.03	0.014
Body weight (kg)	87.6 ± 13	86.6 ± 13	0.001	87.6 ± 13	0.930
Urin Na + 24 h (mmol/day)	216 ± 81	210 ± 17	0.586	222 ± 104	0.660

In contrast, in the placebo group no significant change of tissue sodium content in skin and muscle was observed after 6 weeks of therapy compared to baseline.

Analysing male and female patients separately reduction of skin sodium content in women was numerically higher but without any significant differences (p = 0.400) between the 2 groups (2.00 ± 4.80 A.U.; p = 0.110 vs. baseline in females and 1.01 ± 3.72; A.U.; p = 0.814 vs. baseline in males). Dividing the study cohort on the basis of median age (60.3 years), there was a significant difference in sodium reduction between the two groups; older patients had a lower decrease of skin sodium content than younger patients after 6 weeks therapy with dapagliflozin (0.30 ± 3.75 A.U. vs. 2.50 ± 4.34 A.U.; p = 0.044).

We did not observe any significant changes of muscle sodium content after dapagliflozin therapy when analysing the subgroups categorized by gender or age.

## Discussion

Six weeks of treatment with dapagliflozin improved metabolic control, body weight and reduced office and 24-h ambulatory BP in comparison to baseline in our patients with type 2 diabetes, whereas no change to baseline occurred in the placebo group. In parallel, we found in our exploratory analysis that dapagliflozin was capable of reducing skin sodium content significantly after 6 weeks of treatment, an observation which was not noted in the placebo group.

There was little change in HbA1c on treatment with either placebo or dapagliflozin, which is likely due to the short period of time (6 weeks), in contrast to FPG which was significantly lower after the 6 weeks of dapagliflozin treatment. Dapagliflozin treatment suppresses atherogenic small dense low-density lipoprotein-cholesterol and increases high-density lipoprotein 2-cholesterol [22], [23] and slows the progression of diabetes-associated glomerulosclerosis and liver fibrosis [24]. Furthermore dapagliflozin has previously been shown to reduce BP in patients with type 2 diabetes [25] and improved endothelial function as add-on therapy to metformin to 16 weeks [26], as assessed by flow-mediated dilation in patients with inadequately controlled early stage type 2 diabetes mellitus. This improvement of parameters associated with the early stages of vascular remodelling has been demonstrated repeatedly. Solini et al. showed an acute improvement of endothelial function, reduction of arterial stiffness and renal resistive index in type 2 diabetic patients [27], which is in line with the results of our study group by Ott et al. [19].

In the present study, we observed a decrease in office systolic BP by approximately 4 mmHg and in ambulatory systolic BP by 3 mmHg after 6 weeks of dapagliflozin treatment, which is of a comparable magnitude to those reported in other studies [25]. Body weight was reduced significantly in the dapagliflozin group. Hence, our data indicate an improved cardiovascular risk factor profile as previously described in detail in a real-world primary and diabetologist care setting [28] and also in the CVD-REAL Nordic study, where dapagliflozin treatment was associated with lower risk of cardiovascular events and all-cause mortality in type 2 diabetic patients compared to patients receiving dipeptidyl peptidase-4 inhibitor therapy [29].

The principal finding of this exploratory analysis is that dapagliflozin effected a reduction of sodium tissue content of the skin as measured by ^23^Na-MRI imaging, Skin water content did not change significantly, although skin water content tended to be lower, in accordance with the decrease in body weight. If any, the slight decrease of water skin content may underestimate the observed decrease of skin sodium content after 6 weeks of treatment with dapagliflozin. The decrease of skin sodium content after treatment with dapagliflozin appeared to be small with a 5.8% reduction compared to baseline, but it is totally unknown whether small or large changes are required to alter cardiovascular endpoints in patients with type-2 diabetes. Previously we observed a reduction in skin sodium content after diuretic treatment in patients with acute heart failure [30]. The precise mechanism of decreased sodium content in the skin due to dapagliflozin treatment is unknown. Whether it is a direct effect of dapagliflozin, or a consequence of prolonged decrease in renal sodium excretion, that remains undetectable in the 24-h urinary sampling (due to low sensitivity) [11], remains to be elucidated.

Kopp et al. already showed that hypertensive patients without diabetes have a higher skin sodium content compared to healthy subjects [31]. This higher sodium storage in skin is due to a perturbed signalling mechanism of sodium deposition in skin in hypertensive patients, and represents predominantly non-osmotic storage of sodium [17, 32]. Our finding is in accordance with previous studies that assessed sodium-retention by analysing the exchangeable total body sodium content [33]. Exchangeable sodium content was found to be increased in diabetic patients, in contrast to non-diabetic hypertensive subjects [34]. Of note, as mentioned above, with our ^23^Na-MRI methodology we assess tissue sodium content that is primarily bound to proteoglycan and not osmotically active, thereby representing another consequence of sodium-retention.

In our study skin sodium content in males was higher than in females at baseline, which has already been demonstrated in our previous study [31]. Interestingly reduction in skin sodium content was significantly higher in patients younger than the median age. If this is due to differences in skin composition or explained by a direct stronger effect of dapagliflozin on skin sodium content needs to be further explored. We also plan to obtain more specific information on skin composition by MRI. Nevertheless, since every patient was “his own control”, we think that differences in skin composition should not have influenced our results profoundly.

After initiation of dapagliflozin therapy renal excretion of sodium by blocking SGLT-2 cotransporter [35] is observed but this effect is mitigated or vanishes after 2 weeks of SGLT-2 inhibition [2, 4, 10]. Serum sodium concentration remained stable after 6 weeks treatment with dapagliflozin. In this exploratory study we did not focus on the first 2 weeks, when SGLT-2 inhibition produces natriuresis. We analyzed 24-h sodium excretion during the new steady state after 6 weeks of therapy as an estimate of salt intake. We observed that salt intake as assessed by 24-h sodium excretion, with all its limitations [11], was similar after 6 weeks treatment.

In light of the EMPA-REG Outcome study that showed an early separation of the incidence of hospitalization for heart failure and cardiovascular death between the SGLT-2 inhibitor and the placebo group [6, 36] we want to put forward the hypothesis that whereas the diuretic and natriuretic effect of SGLT-2 inhibition observed in the first days after initiating therapy may acutely decrease preload, even a small natriuretic effect per day may be effected and leads to a reduction of the total skin-sodium content in patients with type 2 diabetes over weeks.

A recent study of Schneider et al. in patients with chronic renal failure, also a sodium retention state as type-2 diabetes, showed the association between increased skin sodium content and increased left ventricular mass [2]. It was demonstrated that skin sodium content is a strong predictor of left ventricular mass, independent of blood pressure, total body hydration and other confounders. These data are in accordance with previous clinical studies describing BP-independent effects of high salt intake on vascular remodelling, left ventricular hypertrophy, cardiovascular events and mortality, and experimental data that found increased sodium content to act as pro-fibrotic and pro-hypertrophic stimuli [5, 37, 38].

## Limitations

Our study has strengths and limitations. The measured sodium content of skin by our current MRI technique has been validated [12]. Full detection of fast relaxing sodium compartments (e.g. sodium in tissue) measured by MRI techniques would ideally require the application of ultra-short echo time pulse sequences [39]. In addition, the low spatial resolution of ^23^Na MRI results in partial volume effects and, thus, may lead to a bias in the ^23^Na MRI skin measurements. Nevertheless a very close relationship between the MRI measured tissue sodium content and experimentally measured tissue sodium content of the amputated parts (in mmol/kg of wet weight) has been found [12]. In future, ^23^Na-MRI of skin might be improved by using a dedicated setup at ultra-high magnetic field strength (e.g. 7 Tesla) [40]. As a consequence our findings need interpretation in context to a direct control group and we compared them to our previous data and to the placebo group obtained with the same ^23^Na-MRI technique [2, 12, 30, 31, 41]. Our results did not show any significant change of tissue sodium content of the muscle. At the moment it is not possible to give a solid explanation for this disparate pattern between the changes of tissue sodium content.

The main limitation of this exploratory study is its short duration of therapy, i.e. we do not know whether the observed effect is maintained over months. This needs to be analysed in future studies (http://www.clinicaltrials.gov; NCT03128528).

## Conclusions

Six weeks of treatment with the SGLT-2 inhibitor, dapagliflozin, resulted in improved diabetic control and reduced 24-h ambulatory BP. While these findings have been demonstrated previously, we showed for the first time that SGLT-2 inhibition was capable of reducing skin sodium content in patients with type-2 diabetes.

Future studies are needed to elucidate and investigate in detail whether reduction of skin sodium leads to improvements of cardiovascular and renal outcomes in diabetic patients that might be mitigated by SGLT-2 inhibition.

## Abbreviations

A.U.: arbitrary unit
BMI: body mass index
BP: blood pressure
CVD: cardiovascular disease
eGFR: estimated glomerular filtration rate
FPG: fasting plasma glucose
^23^Na-MRI: ^23^Na magnetic resonance imaging
SD: standard deviation
SGLT-2: sodium-glucose cotransporter 2
